# IgE Epitope Profiling for Allergy Diagnosis and Therapy – Parallel Analysis of a Multitude of Potential Linear Epitopes Using a High Throughput Screening Platform

**DOI:** 10.3389/fimmu.2020.565243

**Published:** 2020-09-30

**Authors:** Thorsten Krause, Niels Röckendorf, Barbara Meckelein, Heike Sinnecker, Christian Schwager, Stefanie Möckel, Uta Jappe, Andreas Frey

**Affiliations:** ^1^Division of Mucosal Immunology and Diagnostics, Priority Area Asthma and Allergy, Research Center Borstel, Borstel, Germany; ^2^Airway Research Center North, German Center for Lung Research (DZL), Borstel, Germany; ^3^Division of Clinical Molecular Allergology, Priority Area Asthma and Allergy, Research Center Borstel, Borstel, Germany; ^4^Flow Cytometry Core Facility, Institute of Molecular Biology, Mainz, Germany; ^5^Interdisciplinary Allergy Outpatient Clinic, Department of Pneumology, University of Lübeck, Lübeck, Germany

**Keywords:** immunoglobulin E (IgE), allergy diagnosis, allergy therapy, epitope detection, combinatory peptide library, one-bead-one-compound library

## Abstract

Immunoglobulin E (IgE) is pivotal for manifestation and persistence of most immediate-type allergies and some asthma phenotypes. Consequently, IgE represents a crucial target for both, diagnostic purposes as well as therapeutic approaches. In fact, allergen-specific immunotherapy – aiming to re-route an IgE-based inflammatory response into an innocuous immune reaction against the allergen – is the only curative approach for IgE-mediated allergic diseases known so far. However, this requires the cognate allergen to be known. Unfortunately, even in well-characterized allergics or asthmatics, often just a small fraction of total IgE can be assigned to specific target allergens. To overcome this knowledge gap, we have devised an analytical platform for unbiased IgE target epitope detection. The system relies on chemically produced random peptide libraries immobilized on polystyrene beads (“one-bead-one-compound (OBOC) libraries”) capable to present millions of different peptide motifs simultaneously to immunoglobulins from biological samples. Beads binding IgE are highlighted with a fluorophore-labeled anti-IgE antibody allowing fluorescence-based detection and isolation of positives, which then can be characterized by peptide sequencing. Setting-up this platform required an elaborate optimization process including proper choice of background suppressants, secondary antibody and fluorophore label as well as incubation conditions. For optimal performance our procedure involves a sophisticated pre-adsorption step to eliminate beads that react nonspecifically with anti-IgE secondary antibodies. This step turned out to be important for minimizing detection of “false positive” motifs that otherwise would erroneously be classified as IgE epitopes. In validation studies we were able to retrieve artificial test-peptide beads spiked into our library by using IgE directed against those test-peptides at physiological concentrations (≤20 IU/ml of specific IgE), and disease-relevant bead-bound epitopes of the major peanut allergen Ara h 2 by screening with sera from peanut allergics. Thus, we established a platform with which one can find and validate new immunoglobulin targets using patient material which displays a largely unknown immunoglobulin repertoire.

## Introduction

Selectively recognizing foreign matter that has entered the body is a key feature of humoral adaptive immunity. Yet, not always it is clear against which foreign matter an antibody response is directed or with which antigen an antibody will react. A substantial number of asthmatics, for instance, display high total serum immunoglobulin E (IgE) levels but do not react with the common allergens the patients usually are tested for in commercially available routine allergy diagnostic tests (1–3). In the past, those patients were assigned to suffer from “nonallergic asthma” (intrinsic asthma) (4) but recent evidence suggests that those individuals are simply underdiagnosed in terms of allergen reactivity. After all, asthmatics with regular total serum IgE account for less than 6% of asthmatic patients (5). The vast majority of asthmatics display higher total serum IgE. Consequently, including a broader panel of allergens in the testing reveals more cases of “allergic asthma” (extrinsic asthma) among asthmatics (6). So far, many patients with asthma lack proper allergy diagnosis due to the fact that *in vitro* routine allergy diagnostic tests are missing clinically relevant allergen sources, and where allergen sources are included as raw extract allergens, these often lack clinically relevant single allergenic components and, therefore, appropriate sensitivity. Still, allergy diagnostic testing has been vastly improved in the past decades due to molecular allergology providing single allergen molecules, either naturally purified from the source or obtained by recombinant DNA technology (7). The availability of single allergens for singleplex and multiplex assays (component-resolved diagnosis) has already provided the investigators with increased sensitivity, specificity, and diagnostic accuracy of the tests (8, 9). Multiplex assays in microarray format can analyze dozens of potential allergen-specific IgE reactivities in parallel (10–13), thereby allowing fine-profiling of a patient’s sensitization and – together with the clinical history – his/her allergy phenotype. Molecular allergology offers further improvements to diagnostics, pinpointing sensitizations to individual allergen components on the molecular level and providing the basis for refined allergy classifications, risk predictions and personalized treatment regimens (14, 15). Yet, all these diagnostic procedures require the knowledge of at least the primary allergen source. But even nowadays, it often remains an enigma against which allergens the IgE-high-asthmatics actually are sensitized or whether all relevant allergens have been identified as yet.

Therefore, smart approaches are needed in order to determine unknown antibody reactivities and to identify the interaction sites – the so-called epitopes – on the target antigens/allergens. In most cases, these epitopes are protein-derived entities, either linear chains or three-dimensional structures composed of amino acids. A promising line of action consists in offering a broad variety of peptidic targets to the antibody (mixture) in question, and to check for reactivity.

One common approach for identifying peptidic/proteinaceous binding partners for a ligand is the use of phage display libraries (16). Here a pool of DNA sequence motifs is cloned into a permissive site of a phage surface protein. By screening the phage library with the desired ligand(s) potential binders can be isolated, propagated and sequenced in order to reveal the introduced amino acid sequence motif which interacted with the ligand(s). In allergology this technique has been used to study IgE-allergen interaction, either by cloning single chain antibody genes into the phage (17–19) and offering the gene products to a given allergen, or *vice versa* by offering a defined IgE reactivity to a pool of peptide-presenting phages created by cloning random oligonucleotides into the permissive site of the scaffold protein gene (20–23). The latter variant – defined IgE reactivity versus a broad peptide landscape – is more common as it may yield information about the epitope recognized by the IgE in question. Yet, quite often it is necessary to present purified immunoglobulin to the phage library in order to obtain meaningful results (24). This is a clear drawback of this technology as it not only increases the workload but also may cause losses in IgE reactivity due to the purification step.

As an alternative to phage libraries the use of so-called “one-bead-one-compound” (OBOC) libraries constitutes a promising approach. The OBOC technology based on the “split-and-mix” synthesis was invented by Furka et al. (25, 26) and yields a unique peptide species on each bead of the synthesis resin in pico- to nanomolar amounts per bead. Subsequent amino acid sequencing of the bead-bound peptide directly leads to the respective epitope motif. For IgE analysis, an OBOC library has been used once so far, in a study where a known IgE reactivity against shrimp tropomyosin was investigated by screening a broad peptide landscape with sera from shrimp allergics to detect IgE epitopes on tropomyosin (24).

Neither the phage display nor the OBOC technique have been used to reveal epitopes of unknown serum IgE reactivities. We therefore wanted to address this question via the OBOC strategy. In the study presented here, we have developed the methodology for the identification of hitherto unknown IgE reactivities toward unknown allergens via detection of bead-bound linear peptide epitopes.

## Materials and Methods

### Materials

TentaGel S NH_2_ resin was custom-made by Rapp Polymers (Rapp Polymere GmbH, Tübingen, Germany), with the following specifications: approximately 7.1 million polystyrene beads per gram dry powder, 0.36 mmol amino functions/g, bead diameter 60–70 μm under dry conditions, approximately 50 picomoles of functional amino groups per bead. These beads were used as resin material for the on-bead peptide synthesis applying the fmoc solid phase peptide synthesis technique with an automated multiple peptide synthesizer (MultiPep RS, Intavis Bioanalytical Instruments AG, Cologne, Germany).

Monoclonal, humanized IgE antibodies directed against the c-myc epitope as well as anti-human IgE antibodies with different fluorophore labels were obtained from various sources as summarized in Table 1. Human serum was donated by peanut allergic patients in the Allergy Outpatient Clinic of the Medical Clinic Borstel and the Interdisciplinary Allergy Outpatient Clinic, University of Lübeck. Total IgE content and specific IgE reactivity against the peanut allergen Ara h 2 were determined by ImmunoCAP assays (ThermoFisher Scientific/Phadia, Freiburg, Germany). Recognition of linear Ara h 2 epitopes by IgE from patient sera was resolved by in-house epitope mapping analysis as described before (27–30). Use of patient material for this study was approved by the ethics committee of the University of Luebeck (approval number 10-126). All patients gave written informed consent.

**TABLE 1:** Commercially available antibodies used in this study.

Target	Antibody	Label	Source
c-myc	monoclonal IgE, clone 9E10, humanized	–	Absolute Antibody#AB00100-14.0
Human IgE	polyclonal (goat)	DyLight488	Agrisera #AS10758
	polyclonal (goat)	FITC	Nordic-MUBio#GAHu/IgE(FC)/FITC
	polyclonal (swine)	FITC	Nordic-MUBio #SwAHu/IgE(FC)/FITC
	monoclonal, clone BE5	FITC	ExBio #1F-324
	monoclonal, clone 4H10	FITC	ExBio #1F-326
	polyclonal (goat)	DyLight550	Agrisera #AS121901
	monoclonal, clone B3102E8	AlexaFluor555	Southern Biotech#9160-32
	rabbit/human chimeric, Omalizumab	phycoerythrin	Absolute Antibody#Ab00717-23.0
	monoclonal, clone BE5	phycoerythrin	ExBio #1P-324
	polyclonal (goat)	DyLight633	Agrisera #AS122147
	polyclonal (goat)	DyLight650	Agrisera #AS122270
	polyclonal (goat)	DyLight680	Agrisera #AS163319

### Preparation of Polystyrene Beads With Peptides of Defined Sequence

Peptide sequences to be synthesized onto the beads were chosen according to known target structures recognized by human IgE antibodies. As negative control, a scrambled version of each specific peptide was produced with an online tool at http://www.mimotopes.com.

200 mg of the TentaGel S NH_2_ resin (corresponding to approximately 1.5 × 10^6^ beads) were swollen in 5 ml of a 7:3 mixture of dichloromethane (Roth, Karlsruhe, Germany) and dimethylformamide (DMF; Merck Chemicals, Darmstadt, Germany) and transferred in portions of approximately 2.2 × 10^5^ beads (corresponding to 10 μmol amino functions) to 2 ml filter bottom reaction columns (Intavis). In the following, all reagent amounts are given per reaction column. All reaction steps were performed at room temperature. The resin was prepared for synthesis by washing three times with 800 μl of DMF. Fmoc deprotection was achieved by treating the resin two times for 8 min with 400 μl of a mixture of 20% (v/v) piperidine (Sigma-Aldrich, Steinheim, Germany) in DMF and subsequent washing seven times with 750 μl of DMF. Coupling was done by reacting a 10-fold excess of fmoc-protected amino acid building blocks (Merck or IRIS Biotech, Marktredwitz, Germany) with the resin. For this, the resin was incubated two times for 25 min each with a mixture of 77 μl of a 0.6 M fmoc amino acid building block solution in DMF, 25 μl of a 4 M solution of 4-methylmorpholine (Sigma-Aldrich) in DMF and 75 μl of a 0.6 M solution of 2-(1*H*-benzotriazol-1-yl)-1,1,3,3-tetramethyluronium hexafluorophosphate (IRIS Biotech) in DMF. After washing three times with 750 μl of DMF, unreacted amino termini were capped for 5 min with 400 μl of a 5% (v/v) mixture of acetic anhydride (Merck) in DMF. Subsequently the resin was washed and extracted additional six times with 750 μl of DMF.

After synthesis the resin was treated three times for 8 min with 400 μl of a mixture of 20% (v/v) piperidine in DMF, washed seven times with 750 μl of DMF and five times with dichloromethane and dried *in vacuo*. Side chain protecting groups were cleaved off by treatment with 2 ml of cleavage cocktail [92.5% of trifluoroacetic acid (TFA; Roth), 5% of triisobutyl silane (Sigma-Aldrich) and 2.5% of water (all v/v)] for 3 h at room temperature. After incubation, the cleavage cocktail was discarded and the resin beads were washed five times with 10 ml of DMF, five times with 10 ml of pure ethanol (Brüggemann Alcohol GmbH, Heilbronn, Germany), five times with 10 ml of dichloromethane and again five times with 10 ml of DMF. Beads were treated with 30% (v/v) of H_2_O/DMF, 60% (v/v) of H_2_O/DMF and with neat H_2_O for 5 min each at room temperature. Subsequently, the resin beads were washed ten times with 10 ml of phosphate buffered saline (PBS; 2.7 mM KCl, 1.5 mM KH_2_PO_4_, 136 mM NaCl, 8.1 mM Na_2_HPO_4_, pH 7.4), resuspended in 2 ml of PBS containing 0.05% (w/v) of NaN_3_ (final concentration calculated approximately 1.1 × 10^5^ beads/ml) and stored at 4°C. The migration of the beads from organic solvents to an aqueous buffer system results in the swelling of the beads and a final diameter ranging from 90 to 110 μm.

### Synthesis of a Combinatorial/One-Bead-One-Compound (OBOC) Peptide Library via a Manual Split-and-Mix Procedure

**FIGURE 1:**
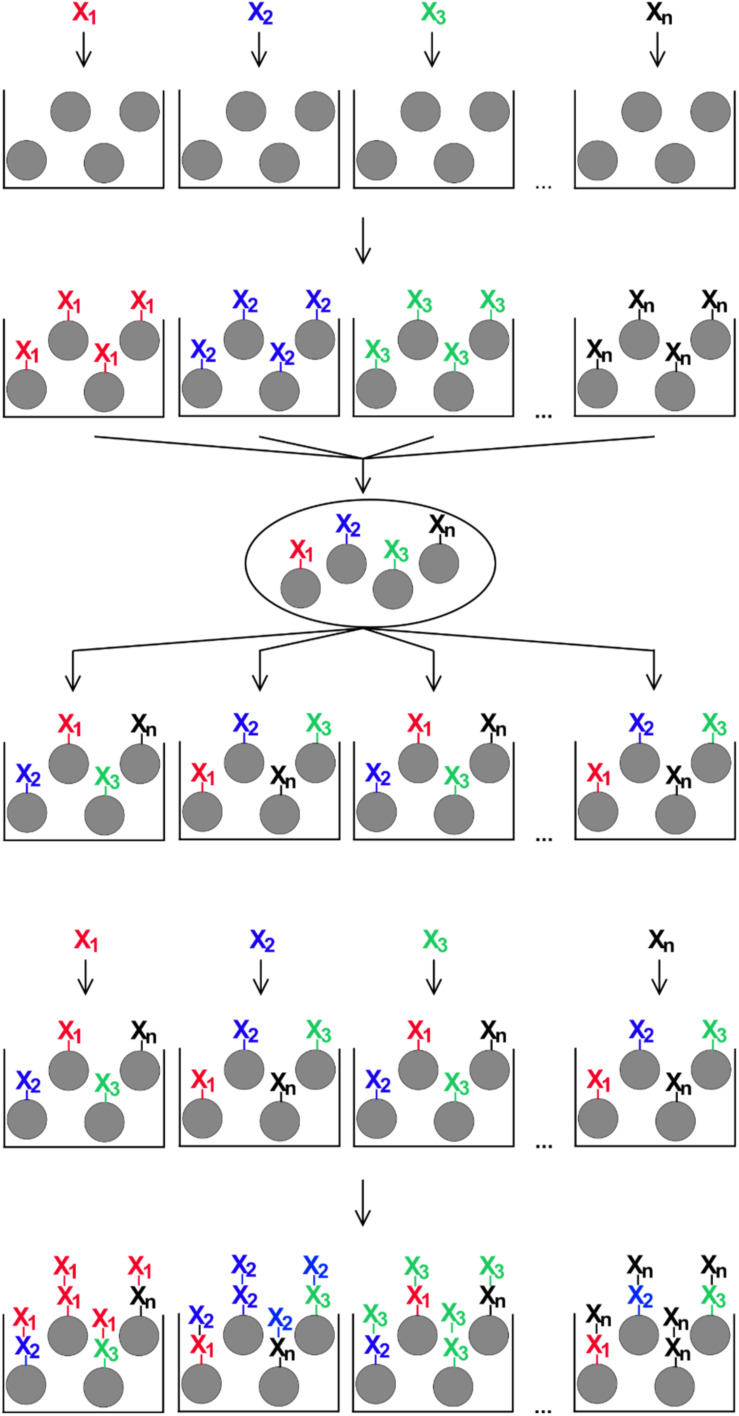
Schematic illustration of the split-and-mix procedure for the generation of OBOC-libraries. For peptide libraries, each of n individual amino acids (X1 – Xn) is coupled to a portion of synthesis resin, then all beads are combined and mixed, distributed again in n portions, and the next coupling step with one individual amino acid per portion is performed. The number of repeated rounds of the procedure corresponds to the peptide length on each individual bead.

OBOC peptides were synthesized based on the procedure published by Lam et al. (31), with some modification to adapt the protocol to our needs. For that, 1.46 g (corresponding to approximately 10 × 10^6^ beads and 0.5 mmol amino functions) of the TentaGel S NH_2_ resin were swollen in 20 ml of a 1:1 mixture of dichloromethane and DMF for 1 h. The resin was distributed equally into 19 polypropylene vials (approximately 0.028 mmol/vial), beads were allowed to settle and the supernatants were removed carefully. To each vial, 433 μl of a 0.6 M solution of one of 19 different fmoc amino acid building blocks in DMF, 433 μl of a 0.6 M solution of 2-(1*H*-benzotriazol-1-yl)-1,1,3,3-tetramethyluronium hexafluorophosphate in DMF and 47 μl of *N*-ethyldiisopropylamine (Sigma-Aldrich) were added. Vials were incubated on an end-over-end mixer at room temperature for 2 h. This way, every proteinogenic amino acid, except cysteine, was coupled to one portion of resin beads. After that, all 19 resin portions were combined in a 20 ml reaction column equipped with a 35-μm filter bottom (Intavis), mixed well, and the supernatant was removed. The resin was washed five times for 2 min at room temperature with 10 ml of DMF each. 10 ml of a solution of 20% (v/v) piperidine in DMF were added and incubated two times for 15 min at room temperature. After that, the resin beads were washed six times for 2 min with 10 ml of DMF, and the resin was again distributed equally to 19 polypropylene vials. This cycle of splitting and mixing the resin beads was repeated eight times to create different random 8mer peptides on the resin beads (Figure 1).

Amino acid side chain de-protection after the last cycle of peptide synthesis, as well as migration of the beads from organic into an aqueous PBS buffer system, were performed as described above for the synthesis of beads with defined peptide sequence. The total OBOC library was suspended in 10 ml of PBS (final concentration calculated 1 × 10^6^ beads/ml) and stored at 4°C after addition of NaN_3_ to a final concentration of 0.05% (w/v), in order to prevent growth of microorganisms.

All bead numbers given in the following protocols are “calculated,” referring to the starting number of beads employed in the respective synthesis. Possible losses occurring during synthesis, washing and handling steps are disregarded.

### Screening of “Artificial” Libraries Consisting of Defined Peptide Sequences

Defined amounts of polystyrene beads carrying specific IgE target peptides (c-myc or Ara h 2, see below) were mixed with the respective scrambled version as irrelevant bead matrix. Ratios of “relevant” to “irrelevant” beads ranged from 1:10 to 1:100.

For screening experiments, mixtures of these beads (corresponding to approximately 10,000 beads in total per assay) were transferred into 2 ml reaction columns (identical to the standard columns used for peptide synthesis; Intavis) with a filter on the bottom of the vessel and a luer outlet. The reaction columns were closed with a luer sealing plug during the incubation of the beads with different reaction solutions. For washing purposes, the sealing plug was removed and the washing buffer was pressed through the beads with a fitting stamp. Contact between the tip of the stamp and the bead-covered filter was carefully avoided to prevent the loss of beads sticking to the stamp’s tip.

The beads were first washed with 2 ml of PBS containing 0.1% (v/v) Tween-20 (Sigma-Aldrich) (PBST) followed by two times washing with the same amount of PBS. After washing, free areas on the polystyrene bead surface were blocked by incubating the beads in 2 ml of blocking solution (0.05% (w/v) fish gelatin (Norland Products Inc., Cranbury, NJ, United States) in PBS) for 1 h at room temperature. Constant mixing of beads in blocking solution was achieved by mounting the tube onto an overhead shaker (Intelli Mixer RM-2L, LTF Labortechnik GmbH) performing a semicircle movement at 10 rpm. This was followed by thorough washing (six times with 2 ml of PBST and two times with 2 ml of PBS). After that, beads were ready for the incubation with the respective IgE antibody preparation used in the specific screening experiment (Table 1). Incubation was done overnight at 4°C on an overhead shaker (semicircle movement at 10 rpm), with IgE concentrations ranging from 1 μg/ml to 1 ng/ml in blocking solution. On the next day, unbound primary antibody was removed by three times washing with 2 ml of PBST and two times washing with 2 ml of PBS. Beads were then incubated with a secondary antibody directed against IgE and labeled with fluorophore (Table 1). Dilutions of the secondary antibodies normally ranged from 1:100 to 1:1,000. In some cases, higher or lower dilutions were used for special experimental purposes. Incubation with the secondary antibody was done at room temperature for 3 h in the dark with 10 rpm of semicircle mixing. Unbound antibodies were subsequently removed from the beads by thorough washing (four times with 2 ml of PBST and four times with 2 ml of PBS). Next, beads were re-suspended in the capped reaction columns using 1 ml of PBS and transferred in portions into 24- or 12-well polystyrene plates (Costar Corning, Corning, NY, United States). PBST was added (usually 5–10% of the total bead suspension volume) to decrease the surface tension of the solution and allow the beads to settle to the bottom of the well.

The beads were examined visually on a standard inverted fluorescence microscope (Nikon ECLIPSE TE2000-U; Nikon, Tokyo, Japan) equipped with a UV light source and filter systems compatible with the fluorophores used. In addition, bead fluorescence intensity and distribution was documented with a MORE life cell imaging microscope (Thermo-Fisher Scientific, Waltham, MA, United States, formerly Till Photonics, Gräfelfing, Germany) equipped with a Clara CCD camera system (Andor Technology, Belfast, United Kingdom), using appropriate filter sets to capture green, red and far red fluorescence signals (FITC-channel: detection filter wavelength: 535 ± 50 nm/rhodamine-channel: detection filter wavelength: 630 ± 75 nm/Cy5-channel: detection filter wavelength: 700 ± 75 nm). Analysis of the respective pictures (including measurement of fluorescence intensities) and image processing was conducted using the software ImageJ v1.52p (NIH, Bethesda, VA, United States) (for details, see Supplementary Material). Visualization of fluorescent beads for photographic documentation and publication was done by converting the 16-bit gray-scale pictures obtained with the respective filters into RGB color space using ImageJ’s built-in look-up tables (LTUs) after fluorescence intensity measurements.

### Separation of IgG and IgE

To separate IgE from IgG, human serum was treated with protein G-sepharose (Ab SpinTrap, GE Healthcare, Chicago, IL, United States) according to the manufacturer’s instructions, with minor modifications. 300 μl of serum from an allergic donor was loaded onto a protein G-sepharose column. The flow-through of the column was collected as IgG-negative/IgE-positive fraction. IgG bound to the sepharose was eluted with 400 μl of acidic elution buffer into a tube containing 30 μl of basic neutralizing buffer, resulting in functional IgG in a buffer of neutral pH.

### Screening of “Artificial” Libraries With Different Human Immunoglobulin Classes

Untreated serum from an allergic donor and the corresponding IgE-fraction obtained as flow through of the protein G-sepharose column were adjusted to the dilution of the eluted IgG-fraction by adding PBS. This results in an approximate 1.4-fold dilution and in an antibody concentration of 70% compared to the original serum. 180 μl of each of these diluted immunoglobulin preparations (total serum, IgE fraction and IgG fraction) were mixed with 20 μl 0.5% (w/v) fish gelatin in PBS, resulting in a final antibody concentration of approximately 65% compared to the untreated serum. These dilutions were then used for the incubation with pre-blocked peptide-bearing polystyrene beads as described above, using a 1:1,000 dilution of the secondary, anti-IgE antibody.

### Screening of OBOC Peptide Libraries Spiked With Defined Peptide Beads

Combinatorial OBOC libraries (either untreated or depleted of anti-IgE cross-reactive beads (pre-cleaned, see below)) were spiked with beads bearing defined peptide sequences (c-myc or Ara h 2; see “Results” section for more information). The ratio “defined peptide bead to OBOC library bead” was between 1:500 and 1:1,000, some preliminary testing was done with ratios of 1:10 to 1:15. The beads were mixed as described above for the “artificial” peptide libraries, and a total of 2 × 10^4^ to 2 × 10^5^ beads were transferred into the reaction column used for screening. The screening protocol was identical to the procedure described for the “artificial” peptide libraries, except for the use of 400 μl of diluted serum/primary antibody and secondary antibody solution (due to higher bead numbers). Antibody concentrations and types of secondary antibodies were varied to identify optimal conditions. After the last washing step, beads in the reaction column were resuspended in 2 ml of PBS, transferred in portions into 12-well or 24-well plates and examined as described above.

### Pre-cleaning of OBOC Peptide Libraries by Pre-adsorption With Fluorophore-Labeled Anti-IgE Antibody and Separation With a Large Particle Sorter

To minimize false-positive results due to binding of secondary, anti-IgE antibody directly to individual beads in the OBOC population, such anti-IgE-cross-reactive beads were identified in a pre-adsorption step and removed from the OBOC library before screening with the actual IgE samples. For this, 1 × 10^6^ beads of an OBOC peptide library were incubated in a reaction column of 20 ml capacity (Intavis) with 15 ml of blocking solution over night at 4°C with semicircle mixing at 10 rpm. Washing steps (before and after blocking) with PBST and PBS were performed as described above, with 15 ml of washing solution per washing step. This was followed by 3 h incubation at room temperature with 3 ml of phycoerythrin-labeled anti-IgE antibody at a concentration of 500 ng/ml and another washing two times with 15 ml of PBST and two times with 15 ml of PBS. Beads were resuspended in a total of 5 ml of PBS and transferred in portions into a 15-ml polystyrene tube. A small sample of the bead suspension was analyzed in the fluorescence microscope to verify successful staining. Afterward, the beads were stored in the 15-ml tube at 4°C in the dark, until separation of fluorescence positive and negative beads was performed, which should be done within 2 weeks after the staining procedure (personal recommendation). This separation was performed on a BioSorter (Union Biometrica, Holliston, MA, United States) equipped with a Fluidics and Optics Core Assembly (FOCA) of 500 μm, using PBS as sheath fluid. Beads were re-suspended in PBS, and concentration was adjusted until a stable event rate of 10–20 events/second was achieved. An unstained bead sample was run as control to set gating conditions. Non-fluorescent, i.e., non-IgE cross-reactive beads (“phycoerythrin-negative”) were sorted with the coincidence mode set to “Pure.” Data graphs were generated in FlowJo software v.10.7.1 (Becton Dickinson, Franklin Lakes, NJ, United States). The fluorescent beads were discarded, the non-fluorescent beads were stored at 4°C until use in screening experiments.

### Pre-adsorption of OBOC Peptide Libraries With Fluorophore-Labeled Anti-IgE Antibody and Screening of Pre-adsorbed Libraries Spiked With Defined Peptide Beads

As an alternative approach to the pre-cleaning step, i.e., the removal of pre-adsorbed anti-IgE-cross-reactive beads from the OBOC-library via BioSorter separation before the actual screening process, the pre-adsorption step was performed using anti-IgE labeled with a different fluorophore than the one used in screening. Afterward, anti-IgE-cross-reactivity was determined in the eventual fluorescence readout.

For this, 2 × 10^5^ beads of an OBOC peptide library, spiked with beads bearing defined peptide sequences (c-myc or Ara h 2) in a ratio of 1:1,000, were incubated in a 3-ml reaction column with 2 ml of blocking solution for 3 h at room temperature under constant mixing. After removal of the blocking solution, 400 μl of fluorescein isothiocyanate (FITC)-labeled anti-IgE (clone BE5; 1:100 in blocking solution) was added, and the incubation was continued for another 3 h. Beads were washed (four times with 2 ml of PBST, two times with 2 ml of PBS) and incubated with 400 μl of anti-c-myc IgE (200 ng/ml) or human serum (160 ng IgE/ml) in blocking solution over night at 4°C under constant mixing. Beads were washed again (three times with 2 ml of PBST, two times with 2 ml of PBS), incubated with 400 μl of phycoerythrin-labeled anti-IgE (clone BE 5; 1:1,000 in blocking solution) for 3 h at room temperature, washed and processed for microscopy as described above.

### Selection and Manual Isolation of Beads

For bead selection and isolation, the standard fluorescence microscope was used. Fractions of the bead mixture which had been examined before in 24- or 12-well plates were transferred into a 6-well plate (Costar Corning) containing 1.8 ml of PBS plus 200 μl of PBST per well. The dimensions of the wells of this plate and the sample dilution allowed access to selected single beads with minimal contact to neighboring beads. This facilitated manual bead manipulation by use of a micropipette. Beads were re-evaluated and adequate beads were chosen for isolation according to their fluorescence properties (for details see section “Results”). Each single chosen bead was removed by using a standard 10 μl pipette with low retention pipette tip. Non-relevant beads were pushed aside and the chosen bead was aspired together with ≤5 μl of the surrounding liquid and transferred into a separate well until further processing for peptide sequencing.

### Peptide Sequencing

After manual isolation of a fluorescence positive bead, the bead was placed onto a trifluoroacetic acid-treated glass fiber disc (Fujifilm WAKO Chemicals Europe GmbH, Neuss, Germany). Location of the bead on the filter was verified via microscopic observation. The filter was loaded into the reaction chamber of an automated peptide sequencing system (PPSQ-53A peptide sequencer, Shimadzu, Kyoto, Japan). Peptide sequences were determined by direct on-bead Edman degradation followed by HPLC separation of the step-wise cleaved-off phenylthiohydantoin-derivatized amino acids. Identification of the individual amino acids was done by reference to a phenylthiohydantoin (PTH) amino acid standard (Fujifilm WAKO Chemicals).

## Results

### Establishing an Assay System to Detect Specific IgE-Binding With Defined Peptides Immobilized on Polystyrene Beads

One-bead-one-compound libraries have the advantage to test millions of peptides in parallel for binding of a defined target molecule. To adapt and optimize this system for the detection of new IgE epitopes, we had to establish an assay format where IgE binding to a specific peptide immobilized on polystyrene beads can be reliably detected. To achieve this, we started with an unambiguous system of a defined IgE antibody – peptide epitope pair in an “artificial peptide library” based on a matrix of irrelevant peptide-carrying beads. We decided on a commercially available, recombinant human IgE antibody directed against the Myc-protein derived peptide c-myc (peptide sequence E-Q-K-L-I-S-E-E-D-L). Beads carrying the c-myc peptide, as well as “irrelevant” matrix beads carrying a scrambled version of the c-myc epitope (sequence E-I-E-D-K-L-S-L-Q-E), were produced by classical fmoc solid phase-based peptide synthesis. These beads were used to optimize assay conditions, especially in terms of anti-IgE antibody employed (type, concentration, fluorophore label), first in order to achieve highest possible sensitivity, and second to take into consideration that the structure of the fluorescent dye coupled to the anti-IgE antibody has an influence on the screening of OBOC libraries (32).

**FIGURE 2:**
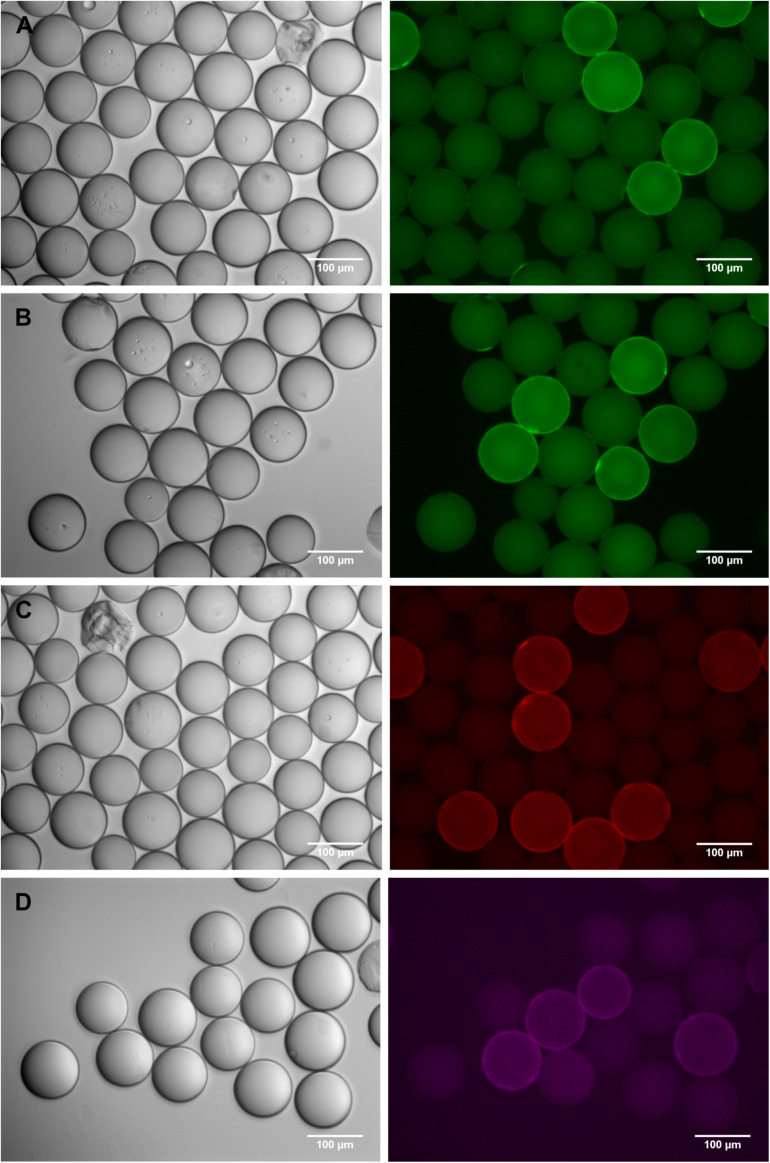
Detection of c-myc-beads, diluted 1:10, in an artificial peptide library of scrambled c-myc beads. Different concentrations of anti-c-myc IgE were used and IgE-binding was detected with various fluorophore-labeled anti-IgE antibodies. Phase contrast images are shown on the left, corresponding fluorescence images of the appropriate channel on the right side of the panel. **(A)** 100 ng/ml anti-c-myc IgE detected with FITC-labeled monoclonal anti-human IgE (FITC-channel); **(B)** 20 ng/ml anti-c-myc IgE detected with polyclonal anti-human IgE labeled with DyLight488 (FITC-channel); **(C)** 20 ng/ml anti c-myc IgE detected with polyclonal anti-human IgE labeled with DyLight550 (rhodamine-channel); **(D)** 2 ng/ml anti c-myc and polyclonal anti-human IgE labeled with DyLight650, (Cy5-channel). High autofluorescence background is predominant in the green channel **(B)**, but true positives can be distinguished by presence of a more brilliant “corona.” Autofluorescence decreases at higher wavelength **(C,D)**. Quantitation of fluorescence intensities can be found in the Supplementary Figure 2.

The first experimental set-up encompassed a 1:10 mixture of c-myc beads and scrambled c-myc beads and an incubation with anti-c-myc IgE antibody in a defined blocking buffer. By successively testing a variety of monoclonal and polyclonal anti-human-IgE antibodies with different green fluorophores (either DyLight 488 or FITC, see Table 1) we were able to improve the assay sensitivity to a detection limit of 20 ng IgE/ml (8.3 IU/ml). Microscopic examination of the results showed that in addition to the intensity, fluorescence distribution can be taken as a criterion for a positive bead, manifested by a distinct fluorescent “corona” at the edge of the bead (Figures 2A,B and Supplementary Figure 2). This is particularly helpful for distinguishing a positive signal from the auto-fluorescence in the green channel which is an imminent drawback of polystyrene beads (33, 34), especially when they bear peptides. In general, this auto-fluorescence is equally distributed across the whole bead area – in contrast to the antibody-derived, specific signal with a fluorescent corona. However, at relatively low signal intensities, the corona is fading and may become invisible over the green auto-fluorescence. In a next step, we therefore wanted to analyze fluorescence-based detection systems with excitation/emission at longer wavelengths, where the auto-fluorescence should be less pronounced.

We used the antibody showing best results in comparative testing (Figure 2B), but equipped with the red fluorophore DyLight 550 (Figure 2C), to detect bead-bound IgE. In this set-up, anti-c-myc IgE at a concentration of ≤10 ng/ml could be shown to specifically label peptide-bearing beads. This was verified by isolation of the fluorescence corona-positive beads followed by peptide sequencing. When the fluorescence was moved even further into the far red range by using (polyclonal) anti-IgE antibodies labeled with DyLight 633, DyLight 650 or DyLight 680 fluorophore, the auto-fluorescence background at the respective far red emission wavelengths was considerably reduced. This improved signal-to-noise ratio allowed us to detect anti-c-myc IgE concentrations as low as 2 ng/ml (0.83 IU/ml) (Figure 2D), which is well in the physiological range of specific IgE in human blood (35, 36). Unfortunately, the far-red fluorescence is barely detectable for the human eye, limiting its application for the manual detection and isolation of fluorescent beads with a standard fluorescence microscope. To facilitate the manual isolation of the fluorescence positive beads, which is an integral part of this platform, we are confident that an optimal trade-off between high microscopic detectability and low auto-fluorescence background may be obtained by using a secondary anti-IgE antibody labeled with a red fluorophore such as DyLight 550 or phycoerythrin.

### Detection of Specific IgE-Epitopes Within a Bead-Bound “Artificial” Library of Defined Peptides Using Serum From Allergic Patients

In addition to establishing our peptide-beads screening assay with the defined recombinant anti-c-myc IgE antibody, we wanted to capture and detect allergen-specific IgE from serum from allergic patients with our bead system. These experiments were in part conducted in parallel to the c-myc experiments described above, hence here too, different secondary antibodies and fluorophores were used.

We employed serum from peanut-allergic patients which had been shown beforehand to have a high specific IgE reactivity against the major peanut allergen Ara h 2 (Table 2). Applying a microarray-based epitope mapping technique (27, 28) we could identify several linear epitopes on Ara h 2 which are recognized by these IgE (data not shown). Based on these mapping experiments, we chose the 8mer R-D-P-Y-S-P-S-P as typical Ara h 2 epitope, which also encompasses two of the three immunodominant Ara h 2 epitopes previously described (37) and which reacted with all sera used by us (three different peanut-allergic donors, Table 2). This 8mer epitope was synthesized on polystyrene beads and “irrelevant” matrix beads again carried a scrambled version of the peptide (P-P-D-R-S-Y-P-S).

**TABLE 2:** Characteristics of patient sera.

Patient ID	Total IgE		Anti-Ara h 2 IgE	
	ng/ml	U/ml	ng/ml	U/ml
P1	3,040	1,267	182	75.8
P2	624	260	53	22
P3	3,880	1,616	>240	>100

**FIGURE 3:**
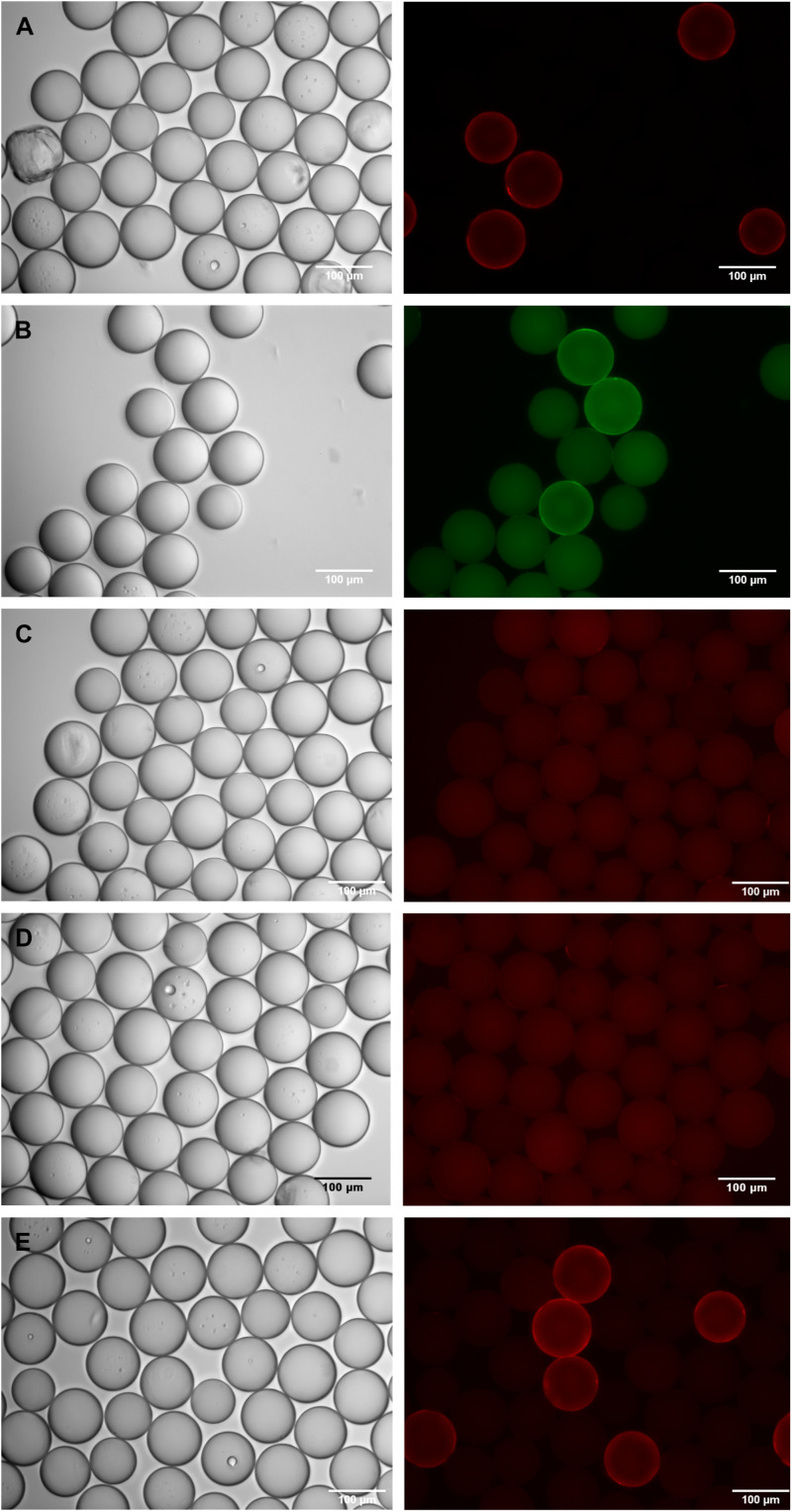
Detection of Ara h 2-beads, diluted 1:10, in an artificial peptide library of scrambled Ara h 2 beads. Beads were incubated with serum from two different peanut-allergic patients, and IgE-binding was detected with fluorophore-labeled anti-IgE antibodies. Phase contrast images are shown on the left, corresponding fluorescence images of the appropriate channel on the right side of the panel. **(A)** Beads incubated with serum from patient 1, diluted to approximately 90 ng Ara h 2-specific IgE/ml; IgE-binding detected with phycoerythrin-labeled monoclonal anti-IgE antibody; **(B)** Beads incubated with serum from patient 1, diluted to approximately 23 ng Ara h 2-specific IgE/ml; IgE-binding detected with DyLight488-labeled polyclonal anti-IgE antibody; **(C)** Beads incubated with total serum from patient 2, diluted to approximately 33 ng Ara h 2-specific IgE/ml; IgE-binding detected with phycoerythrin-labeled monoclonal anti-IgE antibody; **(D)** Beads incubated only with phycoerythrin-labeled monoclonal anti-IgE antibody; **(E)** Beads incubated with IgG-depleted serum from patient 2, diluted to approximately 33 ng Ara h 2-specific IgE/ml; IgE-binding detected with phycoerythrin-labeled monoclonal anti-IgE antibody. Quantitation of fluorescence intensities can be found in the Supplementary Figure 3.

Both bead species were mixed in defined ratios and binding of IgE from human serum from Ara h 2-sensitized peanut-allergic donors was investigated. Even with 1:2, 1:4 and 1:8 diluted serum from one anti-Ara h 2 IgE-positive peanut-allergic donor, Ara h 2 peptide beads could be detected with a green- or red-labeled anti-IgE antibody (Figures 3A,B), and their identity could be verified by manual isolation of the beads and subsequent peptide sequencing.

### Improved Binding of IgE to Its Target Epitope With IgG-Depleted Serum From Allergic Donors

When comparing the sera from different Ara h 2-sensitized peanut-allergic donors in our bead screening assay, we found considerable differences in the fluorescence intensities of the corona-positive Ara h 2 peptide-bearing beads. This is certainly due to the different Ara h 2-specific IgE content of these samples (varying from 50 to ≥240 ng/ml), and to different reactivities against our chosen epitope peptide. A further reason for low IgE detectability may be the presence of IgG with identical epitope recognition in the sample. Along that line, we had realized already from our epitope mapping experiments that we could improve the IgE signals by depleting the serum samples of IgG. Hence, we wanted to port these findings to our bead-based IgE detection, in order to further improve the system.

We therefore compared the bead-based IgE detection of untreated serum with serum which had been depleted of IgG by a protein G-sepharose matrix. We used a serum containing approximately 50 ng/ml of Ara h 2-specific IgE (patient 2), which had shown a specific, albeit not very strong, epitope reactivity in our previous mapping experiments. Yet, the total, untreated serum could not detect any signals above background in our artificial library of Ara h 2/scrambled Ara h 2 peptide beads in combination with a monoclonal, phycoerythrin-labeled anti-IgE antibody (Figures 3C,D). Fluorescence intensities of randomly chosen areas, documented with the MORE life cell imaging microscope and analyzed with ImageJ software, were not different between serum-incubated beads and control beads incubated with only the phycoerythrin-labeled anti-IgE antibody. However, after removal of IgG from this serum, IgE binding to the Ara h 2 peptide positive beads could clearly be detected with this anti-IgE antibody (Figure 3E). This also resulted in a more than threefold increase in the fluorescence intensity measured (see Supplementary Figure 3). Even a serum with a very high anti-Ara h 2 IgE concentration (≥240 ng/ml; patient 3), whose binding to the Ara h 2 peptide beads can be detected by the phycoerythrin-labeled anti-IgE antibody without serum pretreatment, profits from prior removal of the IgG. Here, too, a strong increase in the signal and in the total fluorescence intensity values is observed when the IgE-only fraction is used (data not shown). For both donors the red corona fluorescence-positive beads were verified to be the relevant Ara h 2 peptide beads, via isolation and peptide sequencing.

In addition to enhancing the specific IgE binding, removal of IgG from the serum enabled us to test the anti-IgE antibodies for potential cross-reactivity with IgG. We used the IgG fraction separated from serum of a donor where IgG reactivity with the Ara h 2 epitope had been demonstrated before in our epitope mapping experiments. We incubated these IgG with the artificial Ara h 2 bead library, followed by the monoclonal, phycoerythrin-labeled anti-IgE antibody. Although one can presume, due to our previous data, that IgG-binding to the Ara h 2 beads occurred, no corona-positive red fluorescence was detected, and total fluorescence values were not different from background values. This demonstrates that IgG-cross-reactivity of the monoclonal anti-IgE antibody was negligible.

In total, these experiments show that serum depleted of IgG may improve the detection of target-specific IgE, especially when IgE with only low/medium abundance are analyzed.

### Detection of Specific IgE-Epitopes in One-Bead-One-Compound Combinatorial Peptide Libraries

**FIGURE 4:**
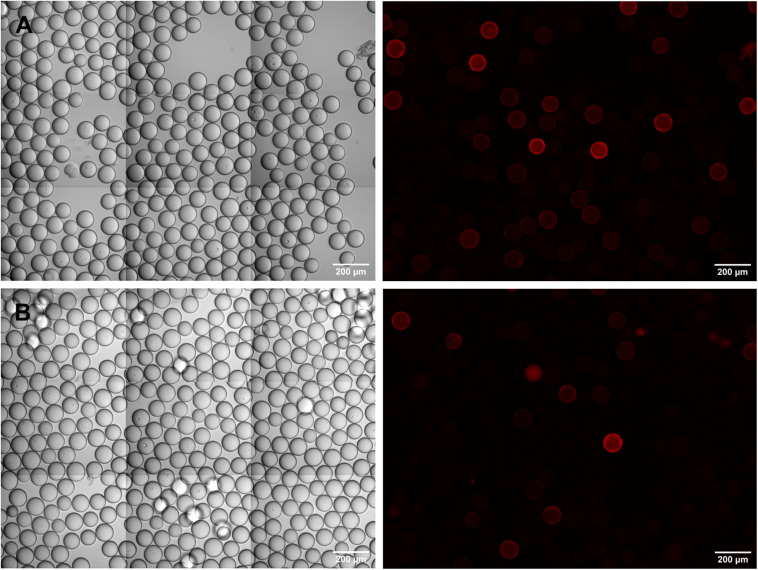
Sporadic cross-reactivity of anti-IgE antibodies with bead-bound random peptides in an OBOC-library. **(A)** DyLight550-labeled, polyclonal anti-IgE antibody; **(B)** phycoerythrin-labeled, monoclonal anti-IgE antibody. Phase contrast images are shown on the left, corresponding fluorescence images on the right side of the panel.

Having established the IgE screening assay with artificial peptide libraries consisting of mixtures of defined peptides, we next moved to the detection of IgE epitopes within a true combinatorial one-bead-one-compound library. An OBOC-library of 8mer peptides was synthesized using the split-and-mix procedure (Figure 1). Portions of the library were spiked with specific c-myc or Ara h 2 beads in defined ratios and screened with anti-c-myc IgE or serum from a peanut-allergic donor. Using a rather small library of several thousands of OBOC beads and a comparatively high number of specific beads (3–5%), we could identify the respective target beads and verify the c-myc respectively the Ara h 2 identity by peptide sequencing (data not shown). However, when increasing the library size and reducing the fraction of specific beads (1:1,000), beads visually identified as being “positive” due to a corona-positive fluorescence were by majority not bearing the specific IgE target peptide when analyzed *via* peptide sequencing. We suppose that this is due to the presence of peptide sequences within the library that directly cross-react with the secondary (anti-IgE) antibody, even without an IgE bound to the beads. In fact, when we incubated the OBOC beads with anti-IgE antibodies alone, without prior addition of IgE or serum, corona-positive fluorescent beads were detectable. This effect was seen – albeit to a varying degree – with all the different anti-IgE antibodies we tested, polyclonal as well as monoclonal, and irrespective of the fluorophore used (Figure 4).

We attempted to suppress this cross-reactivity by variations in the blocking conditions as well as in the secondary anti-IgE antibody concentrations that were used during the screening procedure. Neither of these approaches was successful.

We then tried to block the cross-reactivity by incubating the OBOC library – spiked with specific c-myc or Ara h 2 beads at a ratio of 1:1,000 – with a FITC-labeled anti-IgE antibody at 10-fold the usual concentration. This pre-adsorbed library was then submitted to the standard screening procedure, using anti-c-myc IgE or anti-Ara h 2 IgE-containing serum as primary antibody and a phycoerythrin-labeled anti-IgE antibody for detection. To warrant maximum blocking of cross-reactivity, both secondary antibodies used were derived from the same monoclonal antibody (clone BE5), differing only in their fluorophore label.

**FIGURE 5:**
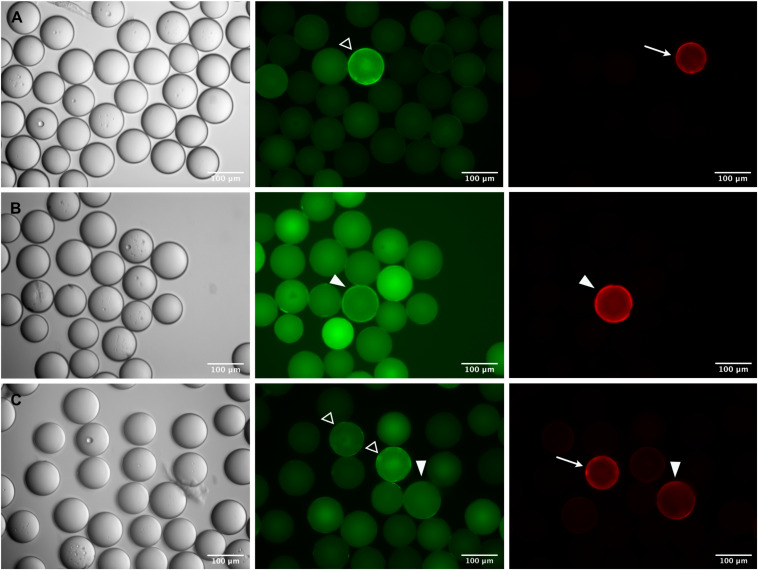
Identification of specific epitope-carrying beads in a pre-adsorbed OBOC library. Specific c-myc- **(A,B)** or Ara h 2- **(C)** beads were mixed 1:1,000 with an OBOC library. Beads were incubated with a FITC-labeled monoclonal anti-IgE antibody to block cross-reactive sequences and subsequently screened with anti-c-myc IgE **(A,B)** or serum from a peanut-allergic patient **(C)** followed by detection of IgE-binding with a phycoerythrin-labeled monoclonal anti-IgE antibody. Phase contrast images are shown on the left, corresponding fluorescence images of the green and the red channel are in the middle and on the right side of the panel. “True positive” beads carrying the specific epitope sequence are recognizable by strong fluorescence only in the red channel (arrows) and only slight autofluorescence in the green channel; pre-adsorbed “cross-reactive” beads show a strong fluorescence with corona in the green channel (open arrowheads), and no signal in the red channel. A number of fluorescent beads cannot be clearly assigned to either “positive” or “cross-reactive,” exhibiting similar fluorescence intensities and/or coronas in both, the red and the green channel (closed arrowheads).

This strategy proved successful in essence, but the parallel use of two fluorophores, and the inherent bead auto-fluorescence problem associated with the green channel, brought about difficulties in the visual inspection and identification of true positive beads. As exemplified in Figure 5, a variety of staining patterns was observed which required interpretation and verification. Basically, fluorescent beads fell into three categories: (1) cross-reactive beads of bright green fluorescence, carrying a corona, which were negative in the red channel (open arrowheads in Figure 5), (2) true positive beads with distinct fluorescence and corona in the red channel and no or only weak (auto-) fluorescence in the green channel (arrows in Figure 5), and (3) questionable beads, where the fluorescence and/or the corona was equally strong in both, the red and green channel (filled arrowheads in Figure 5). The discrimination between the three categories became more difficult if the beads were diluted for manual bead selection. Nevertheless, we were able to isolate single beads from each category and, after sequencing, could verify that beads which had been classified as true positives by their bright red fluorescence were carrying the specific peptides as expected. Questionable beads, on the other hand, which were either less bright in the red channel or also showed up strongly in the green channel, turned out to be of irrelevant sequence. These results demonstrate that the strategy of pre-adsorbing the anti-IgE-cross-reactivities in the OBOC-library before the actual screening process is a feasible approach given the final selection of positive beads is restricted to candidates of strong red and negligible green fluorescence.

### Removal of Anti-IgE Cross-Reactive Beads From an OBOC-Library

**FIGURE 6:**
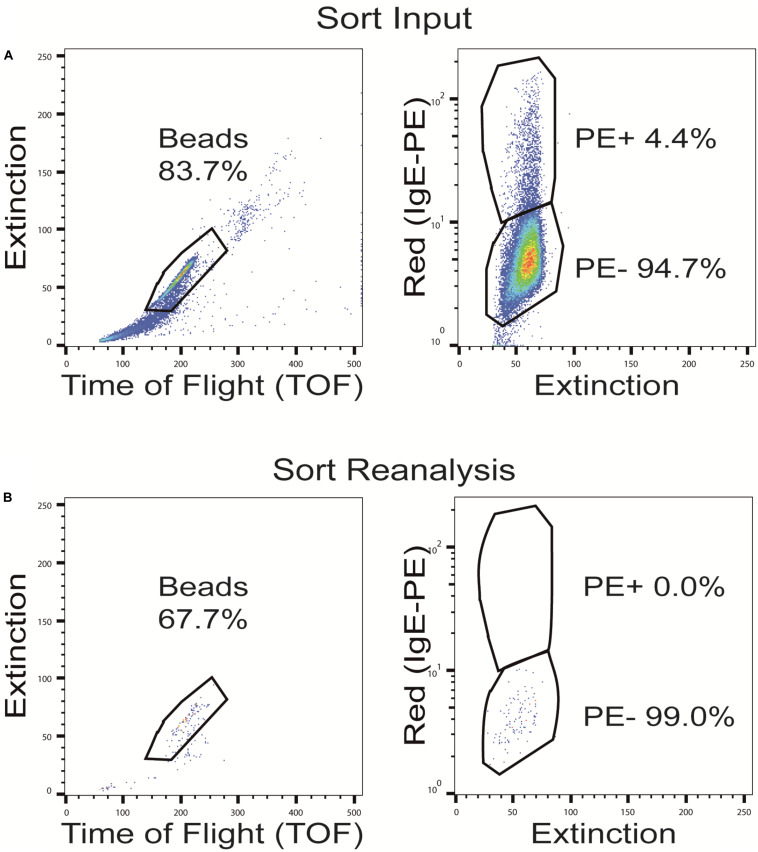
Sorting of OBOC beads after pre-adsorption with the phycoerythrin (PE)-labeled anti-IgE antibody. Representative dot plots with full gating of **(A)** the total pre-adsorbed OBOC library showing the anti-IgE cross-reactive beads in the upper PE+ gate and the non-cross-reactive beads in the lower PE- gate, and **(B)** re-analysis of the pre-cleaned non-cross-reactive bead fraction (PE-) after sorting.

Although our approach to pre-adsorb anti-IgE cross-reactive sequences in an OBOC library before the screening process worked reasonably well, we decided to evaluate another, different strategy to solve the cross-reactivity problem. In this approach, rather than leaving the pre-adsorbed beads within the library and having the green (auto) fluorescence interfere with the subsequent bead isolation process, we wanted to remove the cross-reactive beads from the library pool before the actual screening procedure. To do so, beads of the peptide library were again pre-incubated with high concentrations of a fluorophore-labeled secondary, anti-IgE antibody. Afterward the fluorescence-positive, anti-IgE cross-reactive beads were separated from the fluorescence negative beads (with no intrinsic affinity to the secondary antibody) by sorting with a BioSorter (Figure 6). This procedure resulted in the loss of a fair part of library beads and their potential IgE target sequences (from 2% up to 20%, depending on the individual OBOC library, the secondary antibody used and the exact gating conditions). As a consequence, however, the pre-cleaned library ought not to produce false positive signals due to anti-IgE cross-reactivity, and it does not necessitate the use of a green fluorophore, thereby avoiding the associated auto-fluorescence problems.

### Detection of Specific IgE-Epitopes in OBOC Libraries After Removal of Anti-IgE Cross-Reactive Beads

For our final screening experiments, we used an OBOC peptide library where the anti-IgE cross-reactive beads had been removed via BioSorter separation after treatment with a phycoerythrin-labeled monoclonal anti-IgE antibody. One portion of this pre-cleaned OBOC library was spiked with c-myc beads (ratio c-myc to OBOC = 1:500) and incubated with anti-c-myc IgE (100 ng/ml). A second portion of the pre-cleaned OBOC library was mixed with Ara h 2 beads (ratio Ara h 2 to OBOC = 1:1,000) and incubated with serum from a peanut-allergic donor (specific anti-Ara h 2 IgE = 90 ng/ml). IgE-binding to the beads was detected with the same phycoerythrin-labeled monoclonal anti-IgE antibody that had been used for the pre-cleaning process.

Under microscopic examination, both screening set-ups showed a small number of red fluorescent beads, in line with the low abundance of beads in the library that were carrying specific epitopes. Due to the brightness of the fluorescence, and the low background in the red channel, the individual positive beads could easily be detected and singularized. For each of both set-ups, 10 red-fluorescent beads were isolated and their peptides were sequenced. The correct c-myc sequence could be confirmed for 9 out of the 10 c-myc candidates, and for the Ara h 2 screening set-up, also 9 out of 10 isolated beads could be confirmed to carry the Ara h 2 peptide. Moreover, after repeating the Ara h 2 screening experiment in an identical set-up, but using a different batch of the same monoclonal anti-IgE antibody for detection, 10 of 10 isolated beads carried the Ara h 2 epitope sequence.

In conclusion, we demonstrate here that we have established a method of pre-cleaning and screening an OBOC library that can be used to identify specific IgE-epitope bearing beads within a library of ≥100,000 different peptides.

## Discussion

A considerable number of asthmatics display high total serum IgE levels along with the respective airway pathology but do not react with the typical aeroallergens they are tested for. Although some disorders such as parasite infections or hyper-IgE-syndrome promote the formation of IgE, and atopic predisposition may support class-switch of natural antibodies to class E, it is unlikely that those afflictions account for the high total serum IgE levels that are often associated with asthmatic airway pathology. Parasite infections were shown to protect against asthma (38), cases of hyper-IgE-syndrome are extremely rare (39) and natural antibodies are usually of low affinity (40, 41). However, high affinity seems to be necessary for the pathology that accompanies high IgE levels in allergy and asthma (42). It is thus probable that the high total serum IgE levels in asthmatics are largely composed of IgE which underwent affinity maturation against some specific antigen even if they are negative for the typical aeroallergens. The shortfall of proper allergy diagnosis in such cases is mostly due to the fact that *in vitro* routine allergy diagnostic tests include only a limited number of clinically relevant allergen sources and often lack relevant single allergenic components. *In-vivo* allergy diagnostic tests (skin prick tests) represent the main approach to confirm clinical suspicion of allergic sensitization, but they are mainly based on the application of crude allergen extracts, and standardization remains difficult (43). In this respect, the Global Allergy and Asthma European Network (GA^2^LEN) has established a protocol for a standard prick test panel with a total of just 18 inhalant allergens (43), which they propose to be sufficient for general respiratory allergy testing in Europe. However, considering the more than 3,200 different allergenic molecules identified to date (44) and the often unsatisfactory outcome of allergy testing in high-IgE asthmatics, this test panel appears to be all too limited for a personalized diagnostic approach.

Anyhow, as soon as an allergy could be proven to be the initiator of asthma, and the corresponding allergen could be identified, causative treatment strategies (like allergen-specific immunotherapy) can be introduced to the respective patients. Thereby a specific anti-inflammatory treatment can be provided and – if necessary – can be combined with treatment of disease-relevant target molecules (target treatments), like the application of biologicals against certain cytokines (45). In any case, the patients will profit considerably from an improved molecular allergy diagnostic test based on specifically recognized epitopes.

In principle, the specificity of high affinity IgE can be deduced from their selective recognition of cognate allergen or allergen epitope upon its being offered for binding. Unfortunately, most bioinformatical approaches to predict an epitope on the basis of the paratope/CDR sequence (46, 47) did not meet up to the expectations as yet. Hence, a procedure where an abundance of potential epitopes can be offered to an IgE population with unknown specificities appears to be more promising. Although phage display libraries may be an apparent choice in this context, the presentation of an epitope in a permissive scaffold phage protein may be futile. An epitope’s presentation in the original topology of its parent allergen is usually indispensable for recognition, and restricted epitope flexibility may prevent induced-fit-binding. On the other hand, the structural constraints and rigid presentation of the peptide insert as part of the scaffold protein render phage display libraries more suited to identify mimotopes that imitate the structure of a conformational epitope (48). In such a case, the amino acid sequence of the peptide insert does not necessarily have any resemblance to that of the natural IgE epitope, and deductions as to the respective antigen/allergen recognized are not feasible. Thus, a display system such as chemically synthesized peptides flexibly linked to the surface of microparticles, where an epitope is free to adopt any conformation, may be more advantageous to identify a linear epitope or the linear core motifs of conformational epitopes. We therefore decided to adapt the OBOC technology for our scientific research question.

Any library, OBOC as well as phage display, is limited by the number of peptides which can be presented. A comprehensive 8mer motif library will contain 1.7 × 10^10^ different permutations when 19 amino acids are used. This translates into about 2 times 10 billion different phages or 240 kg of OBOC beads. Such huge numbers of phages and amounts of beads are impossible to handle and to screen. Yet, typical epitopes contain a core region of 4–7 amino acids (mean 5.5) (49, 50) which translates into roughly 10 million possible permutations. In view of this, in our OBOC-library of 10 million beads all relevant core motifs should theoretically be contained in the carboxy terminal part (that is the first 5–6 amino acids) of all peptides as a whole. The closer to the amino terminus we get, the more permutations are not represented. We nevertheless decided to extend the OBOC library toward being “non-representative” by using 8mer peptides in order to further increase our chances to identify epitopes which require more than the minimal core region for recognition. We consider this length a good compromise between being representative with the number of beads that can be handled, and the chances of displaying epitopes sufficiently long to be specifically recognized.

In order to establish a robust screening procedure that uses OBOC-libraries for epitope discovery, we decided for a stepwise, systematic approach to solve any intrinsic and extrinsic problems. The main problem to be overcome turned out to be the necessity for high sensitivity of the assay system in order to enable us to detect a specific reactivity with IgE at physiological concentrations. In healthy people the total serum IgE level is usually below 100 IU per ml. A total serum IgE value ≥100 IU/ml (corresponding to 240 ng/ml) is considered indicative of allergy/atopy, and in some allergic patients serum IgE against one specific target may reach 1,000 IU/ml (2,400 ng/ml) and above. Still, these immunoglobulin concentrations are in a range where detecting their specific reactivities on a one-bead-level may become a problem. Addressing the sensitivity issue, we tested a variety of different reporter systems attached to anti-IgE antibodies to visualize binding of IgE to the beads. Initial attempts with enzymatic reporters that produce insoluble dyes failed to provide the desired sensitivity and/or specificity, even when tyramide signal amplification (51) or fluorescent substrates such as Amplex Red (52) were used. We therefore switched to fluorophore labels directly conjugated to the secondary antibody, additionally providing the possibility to detect, (pre-) sort and isolate specific beads automatically via fluorescence properties with cell sorter techniques. While this system proved more sensitive, we encountered another difficulty associated specifically with fluorescence detection. The polystyrene-based OBOC bead matrix, in particular when covered with peptide, is not optically inert (33, 34). It tends to display a considerable auto-fluorescence at green emission wavelength (535 ± 50 nm), which obscures the specific signal when e.g., FITC is used as label. We therefore decided to move to reporter fluorophores with higher emission wavelength. Here bead auto-fluorescence was considerably lower and detection sensitivity higher. The sensitivity benefit of migrating toward near infrared was, however, limited by the capacity of the human eye to detect far red light since the beads are being picked manually under visual inspection. For the future, we envisage that the whole screening and bead-isolation process might be automated in a microfluidic system. If this is equipped with an infrared camera the use of near infrared dyes such as DyLight755 or DyLight800 becomes feasible and detection limits can be extended beyond the limits reported in this study.

A further problem which we had not initially anticipated was the capacity of certain beads in the OBOC library to directly acquire secondary antibody. Although the extent of direct binding to the beads varied with the secondary antibody(-conjugate) used, it appears likely that the main determining factor for this cross-reactivity resides in the peptide sequence motifs presented on specific beads. This includes peptides which by chance resemble part of the IgE heavy chain or motifs that have affinity to some region of the secondary antibody or of the fluorophore. Analysis of such undesired binders revealed that they mostly contain aromatic amino acids and proline. Similar results were reported in another study, where OBOC beads were screened with a probe equipped with the fluorophores ATTO590 and TexasRed. Here, the amino acids leucine/isoleucine, histidine, phenylalanine and tyrosine were enriched in false positives reacting with the dye (32). These data are intriguing. Yet, when considering that in an OBOC library where all amino acids are used in equal amounts during synthesis, the three aromatic amino acids as well as histidine are overrepresented as compared to their natural frequency observed in proteins (53), the undesired binders may at least be explainable. Unfortunately, little information is available about amino acid distributions in IgE binding sequences. So far only one study (54) addressed this question and reported a preference for Ala, Ser, Asn, Gly and especially Lys in IgE epitopes. In light of this, it may be advisable to create “natural amino acid frequency-representing” OBOC libraries where the occurrence of certain amino acids reflects the natural occurrence of those building blocks. Further restrictions, on the other hand, such as overweighing the amino acids preferentially found in IgE epitopes (54) may limit the diversity of the OBOC library offered.

In order to reduce undesired direct secondary antibody labeling of beads without tampering with library composition we followed two avenues of resolution for the problem, both of which proved practicable. In the first approach, the complete OBOC-library was pre-incubated with an excess of secondary antibody equipped with a green fluorescent dye, then incubated with the IgE pool and finally exposed to the same secondary antibody labeled with a red fluorophore. In this case the decision between false and true positives has to be made by comparing green and red fluorescence of individual beads upon microscopic inspection. In practice, this turned out to be a rather time-consuming process with a certain operator variability. Focusing on beads with a strong corona in the red fluorescence channel and negligible green fluorescence yielded an excellent true-positive rate – as verified by sequencing – in our experimental set-up. However, it must be surmised that the same strategy decreases the sensitivity of the procedure and reduces the probability to positively identify epitopes of rare IgE species. It therefore seemed reasonable to remove any cross-reactive beads from the OBOC library before the actual screening process. This was achieved by sorting the fluorescently pre-adsorbed OBOC library with a large particle sorter (BioSorter). While this approach led to satisfactory results in our set-up, it must be conceded that the limited availability of suitable sorting devices renders this procedure not highly practicable for general use. Here certainly remains room to improve the protocol, either by adaption of more accessible sorting devices (e.g., FACS) to the specific particle size used in the libraries, or by refining the back-up protocol employing two differently labeled anti-IgE antibodies with the use of fluorophores less sensitive to the interfering bead auto-fluorescence.

The final step to validate our OBOC library screening procedure was the verification of the true positives after bead isolation by peptide sequencing. First attempts using mass spectrometric analysis (MALDI-TOF-MS) of fragments after ammonolysis with aqueous or neat ammonia, however, were not successful. Although some studies report mass spectrometrical sequencing of peptides released from beads upon ammonolysis (32, 55–57), in our hands OBOC beads did not liberate equal amounts of cleavage product upon ammonia exposure which caused difficulties to reconstruct the synthesized motif *in silico*. Even known peptide sequences on defined c-myc or Ara h 2 beads could not be resolved by MALDI-TOF-MS. We therefore switched to Edman degradation-based peptide sequencing on an automated protein sequencer. Single isolated beads were directly subjected to the sequencing process, without the necessity of first removing bound antibodies or cleaving the peptide off the bead. Although the low amount of peptide present for sequencing (maximum theoretical capacity per bead 50 pmoles) is close to the sensitivity limit of the sequencer, the peptide sequence on the isolated beads could be successfully resolved in the great majority of cases. Furthermore, beads with higher peptide capacity (e.g., 100 pmoles) are available. All procedures described here could be adapted without much effort to those beads, in case of ambiguous results in terms of peptide sequencing during OBOC library screening.

Taken together, we here present a detection system to identify unknown IgE reactivities by using chemically synthesized one-bead-one-compound libraries. We are confident that this technology will aid in the identification of novel allergens for asthmatic individuals with high total serum IgE and with no specific allergic reaction detectable to date.

## Data Availability Statement

The raw data supporting the conclusions of this article will be made available by the authors, without undue reservation.

## Ethics Statement

The studies involving human participants were reviewed and approved by Ethics Committee of the University of Lübeck. The patients/participants provided their written informed consent to participate in this study.

## Author Contributions

TK designed the study, designed and performed the experiments, analyzed and interpreted the data, and wrote the manuscript. NR and BM designed and performed the experiments, analyzed the data, and wrote the manuscript. HS, CS, and SM performed the experiments, analyzed the data. UJ recruited and characterized peanut-allergic patients, collected the samples, and discussed the data. AF conceived and supervised the study, discussed the data, and wrote the manuscript. All authors read, revised and approved the manuscript for submission.

## Conflict of Interest

The authors declare that the research was conducted in the absence of any commercial or financial relationships that could be construed as a potential conflict of interest.
