# Supplementary material for: IgE Epitope Profiling for Allergy Diagnosis and Therapy – Parallel Analysis of a Multitude of Potential Linear Epitopes Using a High Throughput Screening Platform
Source: Front Immunol. 2020 Sep 30;11:565243. doi: 10.3389/fimmu.2020.565243 (PMC7561404; doi:10.3389/fimmu.2020.565243)
Supplement: Supplementary file 1 [file Data_Sheet_1.PDF]

Quantitative analysis of bead fluorescence:

For quantitative analysis of bead fluorescence, pictures of beads were captured with a MORE life cell imaging microscope (Thermo-Fisher Scientific, Waltham, MA, USA, formerly Till Photonics, Gräfelfing, Germany) equipped with a Clara CCD camera system (Andor Technology, Belfast, Northern Ireland), using appropriate filter sets for the green, red and far red fluorescence signals (FITC-channel: detection filter wavelength: 535±50 nm / rhodamine-channel: detection filter wavelength: 630±75 nm / Cy5-channel: detection filter wavelength: 700±75 nm). For each individual experiment, 2-5 pictures from different regions of the respective well were taken, with the magnification set at 10x. Analysis of the respective pictures (including measurement of fluorescence intensities) and image processing was conducted on 16 bit grayscale images, using the software ImageJ v1.52p (NIH, Bethesda, VA, USA) .

To compare total fluorescence intensities of "positive" and "negative" beads in ImageJ, minimum and maximum threshold values were set in each picture to define bead populations, and mean fluorescence intensities of respective areas ("mean population fluorescence") were calculated (see Supplementary Figure 1 below).

For quantification / documentation of single bead fluorescence, 4-8 individual beads were chosen in a picture, a diagonal line (length 240 pixel) was drawn across the respective bead, and the relative fluorescence intensity (gray scale value) along this line was recorded pixel-wise. For visualization of fluorescence intensities and "corona effects", data were processed using GraphPad Prism version 5.0f (GraphPad Software Inc., San Diego, CA) (see Supplementary Figures 2 & 3 below).

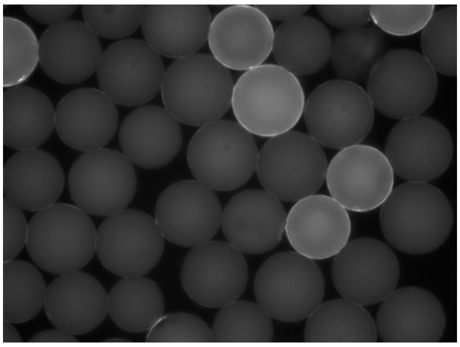

	Area	Mean	Min	Max	MinThr	MaxThr
1	169161	2257.385	1994	4416	1994	4490
2	1025929	1643.111	1395	1994	1395	1994
3	245766	1183.240	867	1380	0	1380

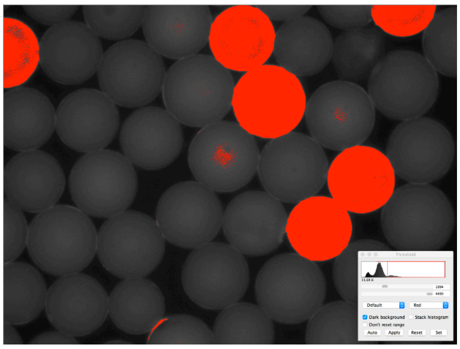

Threshold setting 1: „positive beads“
(fluorescence intensities: 1994-4490)

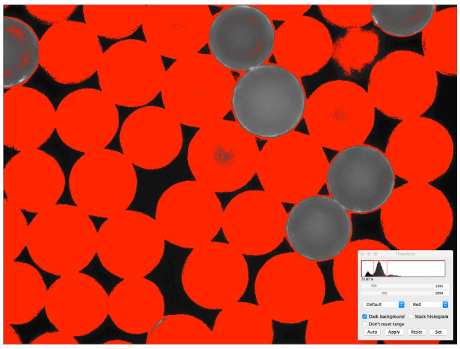

Threshold setting 2: „negative beads“
(fluorescence intensities: 1395-1994)

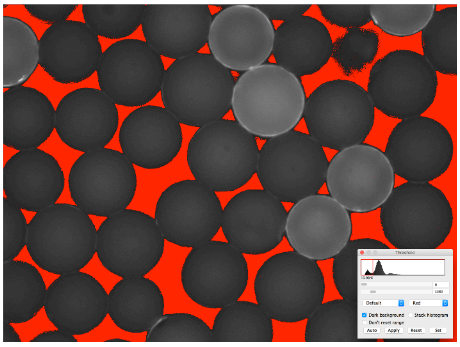

Threshold setting 3: „background“
(fluorescence intensities: 0-1380)

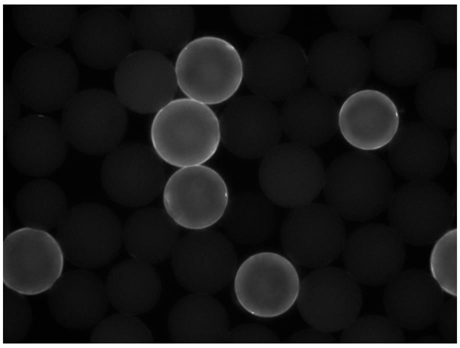

	Area	Mean	Min	Max	MinThr	MaxThr
1	196849	943.435	807	1976	807	2005
2	1204260	670.923	602	801	602	801
3	82827	599.367	543	608	0	608

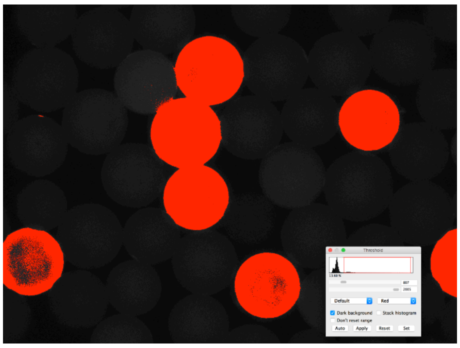

Threshold setting 1: „positive beads“
(fluorescence intensities: 807-2005)

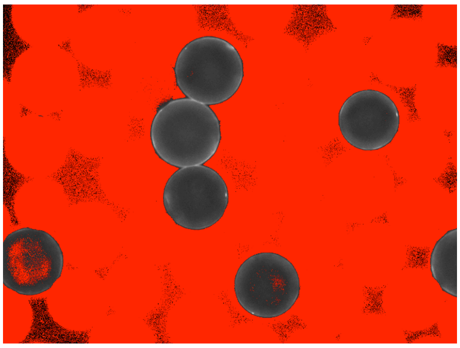

Threshold setting 2: „negative beads“
(fluorescence intensities: 602-801)

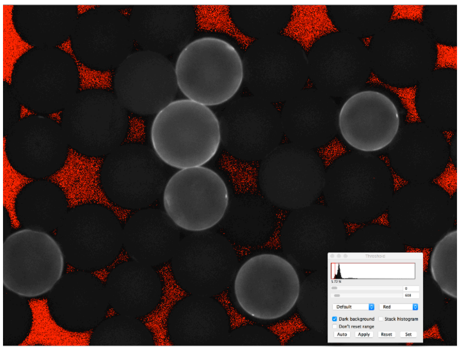

Threshold setting 3: „background“
(fluorescence intensities: 0-608)

Supplementary Figure 1: Examples of threshold-settings for quantitative analysis of fluorescence intensities of beads („mean population fluorescence“): Upper part: library of c-myc-beads, diluted 1:10 with scrambled c-myc-beads, and detected with anti-c-myc IgE and FITC-labeled anti-IgE antibody (see Figure 2A in Original manuscript); lower part: library of Ara h 2-beads, diluted 1:10 with scrambled Ara h 2- beads, and detected with IgG-depleted patient serum and phycoerythrin-labeled anti-IgE antibody (see Figure 3E in Original manuscript). Areas corresponding to fluorescence intensities within chosen thresholds (population 1: “positive“, population 2: „negative“, 3: „background“) are highlighted in red.

A: primary antibody: 100 ng/ml anti-c-myc IgE
secondary antibody: anti-IgE-FITC

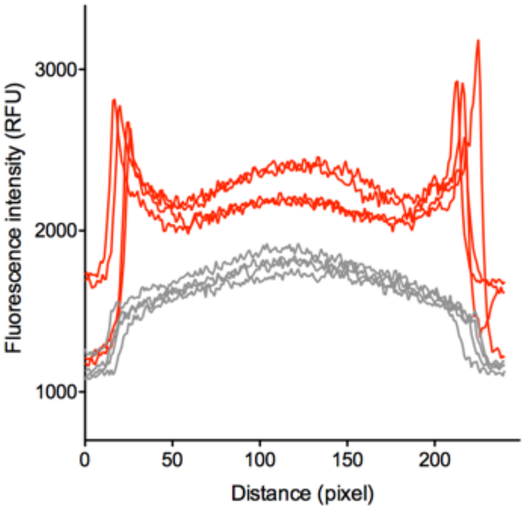

Positive	Negative	Positive - background	Negative - background	Ratio P/N
2257	1643	1074	460	2.33

B: primary antibody: 20 ng/ml anti-c-myc IgE
secondary antibody: anti-IgE-DL488

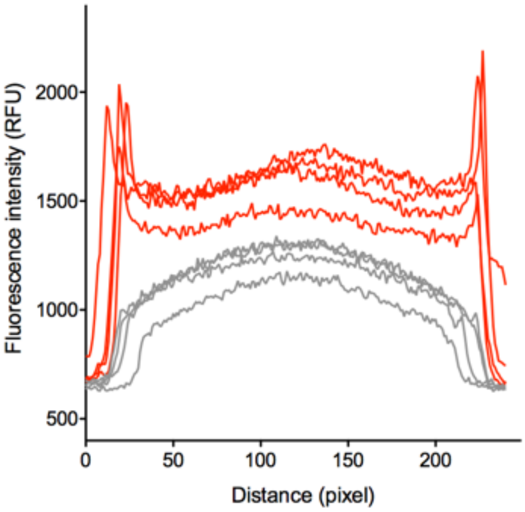

Positive	Negative	Positive - background	Negative - background	Ratio P/N
1544	1155	895	507	1.77

C: primary antibody: 20 ng/ml anti-c-myc IgE
secondary antibody: anti-IgE-DL550

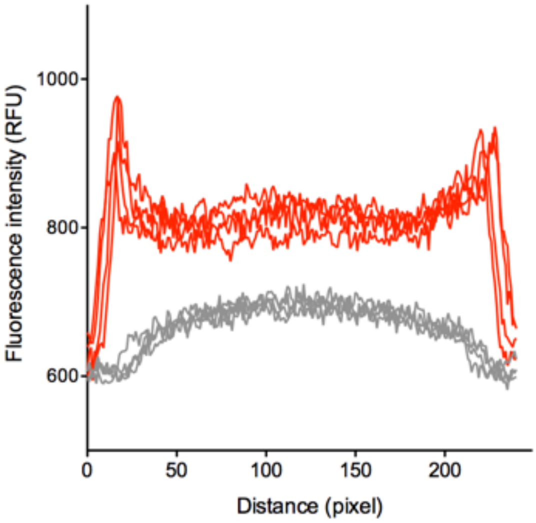

Positive	Negative	Positive - background	Negative - background	Ratio P/N
817	680	204	66	3.09

D: primary antibody: 2 ng/ml anti-c-myc IgE
secondary antibody: anti-IgE-DL650

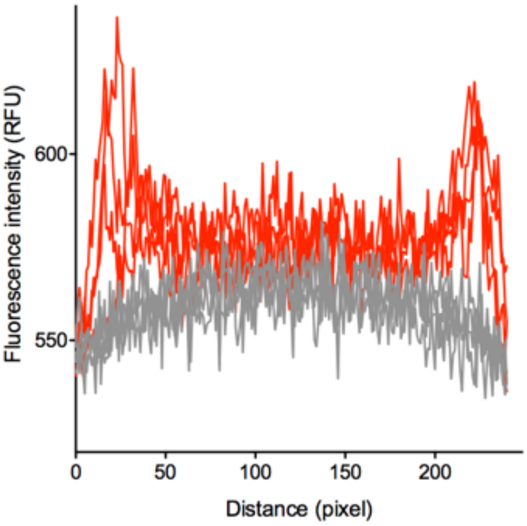

Positive	Negative	Positive - background	Negative - background	Ratio P/N
586	566	48	28	1.70

Supplementary Figure 2: Single bead fluorescence measurements and mean population fluorescence quantification of beads: Library of c-myc-beads, diluted 1:10 with scrambled c-myc-beads, and detected with anti-c-myc IgE and different fluorophore-labeled anti-IgE antibodies. **Quantification of experiments corresponding to figure 2 A-D in original manuscript.**
Graphs show fluorescence intensities (corresponding to gray values of the 16-bit gray scale image) of 240-pixel cross-sectional cuts of „positive“ (red lines) and „negative“ (grey lines) beads. Note increased fluorescence at borders of positive beads indicating the corona.
Tabular data show mean fluorescence values without/with background substraction and signal ratios of „positive“ and „negative“ beads.

A: primary antibody: patient serum #1, 90 ng/ml anti-Ara h 2 IgE
secondary antibody: anti-IgE-phycoerythrin

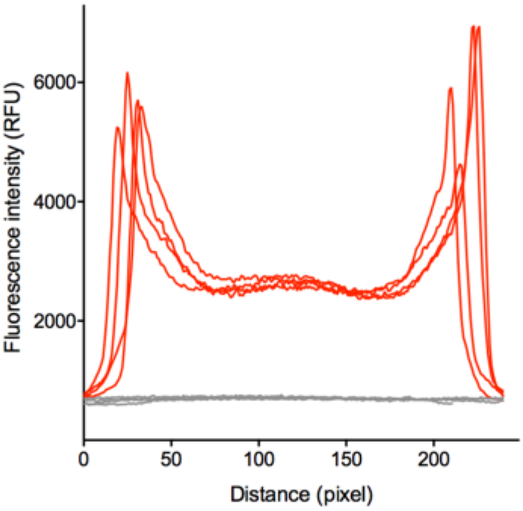

Positive	Negative	Positive - background	Negative - background	Ratio P/N
3204	702	2592	90	28.67

B: primary antibody: patient serum #1, 23 ng/ml anti-Ara h 2 IgE
secondary antibody: anti-IgE-DL488

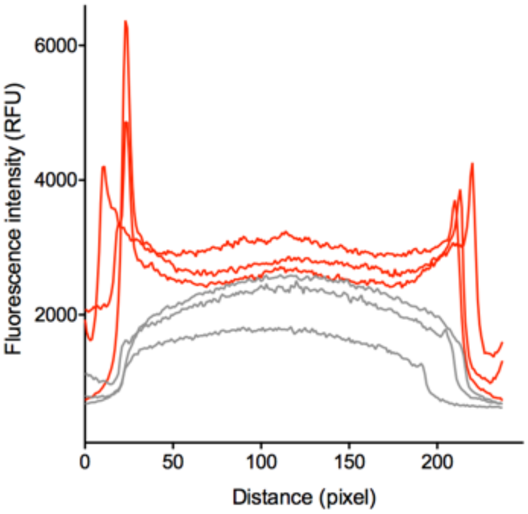

Positive	Negative	Positive - background	Negative - background	Ratio P/N
2883	1963	2219	1298	1.71

C: primary antibody: patient serum #2, 33 ng/ml anti-Ara h 2 IgE
secondary antibody: anti-IgE-phycoerytrhin

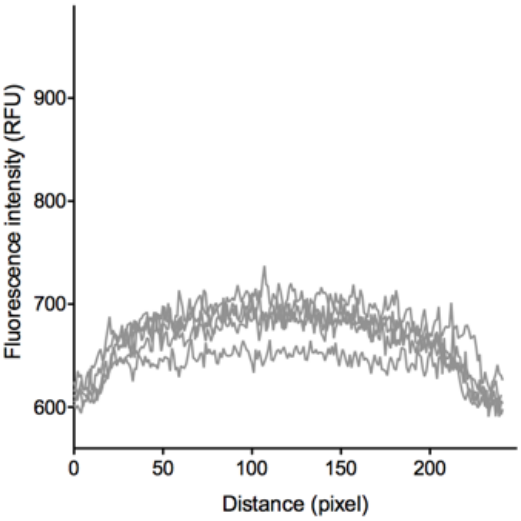

Positive	Negative	Positive - background	Negative - background	Ratio P/N
--	670	--	66	n.a.

D: no primary antibody
secondary antibody: anti-IgE-phycoerytrhin

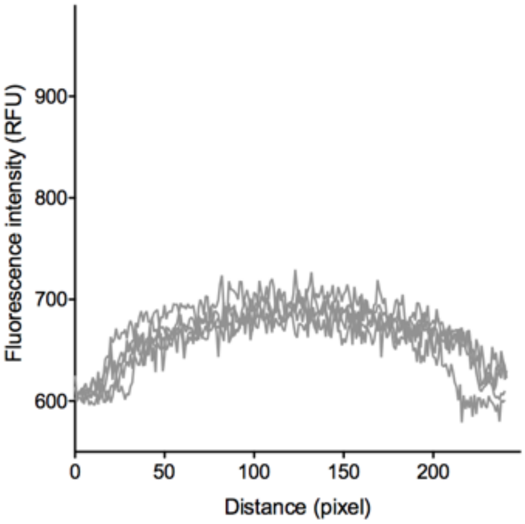

Positive	Negative	Positive - background	Negative - background	Ratio P/N
--	670	--	64	n.a.

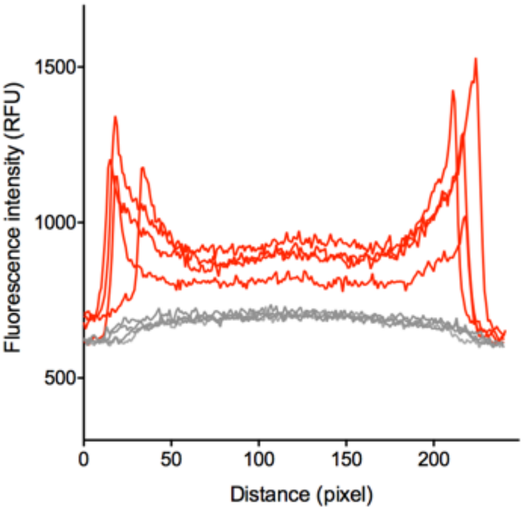

E: primary antibody: patient serum #2, 33 ng/ml anti-Ara h 2 IgE, after IgG depletion
secondary antibody: anti-IgE-phycoerytrhin

Positive	Negative	Positive - background	Negative - background	Ratio P/N
943	671	374	72	5.23

Supplementary Figure 3: Single bead fluorescence measurements and mean population fluorescence quantification of beads: Library of Ara h 2-beads, diluted 1:10 with scrambled Ara h 2-beads, and detected with sera from peanut-allergic patients and different fluorophore-labeled anti-IgE antibodies.
Quantification of experiments corresponding to figure 3 A-E in original manuscript.
Graphs show fluorescence intensities (corresponding to gray values of the 16-bit gray scale image) of 240-pixel cross-sectional cuts of „positive“ (red lines) and „negative“ (grey lines) beads. Note increased fluorescence at borders of positive beads indicating the corona in panels A, B and E.
Tabular data show mean fluorescence values without/with background substraction and signal ratios of „positive“ and „negative“ beads.
Panel C shows no „positive“ bead population with fluorescence signals above „negative“ beads due to anti-Ara h 2-IgG reactivity in patient serum #2 which interferes with IgE binding and decreases sensitivity of IgE-detection.
Panel D shows reactivity of secondary antibody only, hence no „positive“ bead population is detected, only „negative“ beads.
